# Synergistic Catalysis of Co(OH)_2_/CuO for the Degradation of Organic Pollutant Under Visible Light Irradiation

**DOI:** 10.1038/s41598-020-59053-9

**Published:** 2020-02-06

**Authors:** Naeem Akram, Jia Guo, Wenlan Ma, Yuan Guo, Afaq Hassan, Jide Wang

**Affiliations:** Key Laboratory of Oil and Gas Fine Chemicals, Ministry of Education & Xinjiang Uygur Autonomous Region, College of Chemistry and Chemical Engineering, Xinjiang University, Urumqi, 830046 China

**Keywords:** Photocatalysis, Environmental chemistry, Nanoscale materials

## Abstract

The exploration of advanced water treatment technologies e.g. heterogeneous photocatalysis is the most promising way to address organic pollution issues. Semiconductors based bimetallic photocatalysis with wide bandgap, have displayed splendid degradation performance in the UV light region, but their extension to the visible light/near infra-red region is still a matter of great concern. CuO, Co(OH)_2_, CoO and Co(OH)_2_/CuO nanocomposites were synthesized via simple co-precipitation method and further practiced for Rhodamine B (RhB) decomposition by introducing per-sulfate (PS) as a sacrificial agent. Results revealed that Co(OH)_2_/CuO catalyst had shown robust catalytic activity for RhB photodegradation (degradation time 8 min, k = 0.864 min^−1^) under light illumination, significantly less (12–60 times) than the other reported bimetallic catalysts. Catalyst also have verified excellent performance for a broader pH range (5–9) with excellent stability. Main reactive species responsible for the photocatalytic reaction were sulfate (SO_4_^•−^) and superoxide (O_2_^•^) radicals, duly verified by ESR and by using radical scavengers. With outstanding recycling abilities, this is probably the fewer successful attempt for RhB decolorization and can be highly favorable for effluent treatment by using the synergic effect of absorption and photodegradation.

## Introduction

Globally increased population resulting in escalating environmental pollution, particularly, water contamination is consistently grabbing attention of researchers to overcome the needs of clean and safe water sources, for many decades^1^. Ever-increasing garbage and effluents of many industries especially the usage of synthetic dyes in textile, tannery, paint, and paper industries are continuous sources of contaminated water^2^. Releasing these dyes into the water sources, affecting aquatic life, ecosystems and human beings, adversely^3^. Thus, the exploration of new and effective ways to overwhelmed organic water pollution issues is always considered as challenging. Many biological, physical, chemical, electrical and electrochemical methodologies have been developed, so far, for the removal of organic matter^4^. From these methodologies, advanced oxidation processes (AOPs) have been extensively employed because of their simple, efficient and feasible approach. Amongst AOPs, heterogeneous photocatalysis has remarkable potential for organic pollutant removal, also has eco-friendly behavior, good stability, efficient photocatalytic activity for various dyes and most advantageously their economical and simple synthesis^5^. Many techniques including sol-gel^6^, co-precipitation^7^, soluble sacrificial salt template^8^, solvothermal^9^ and microwave-assisted approach^10^ have been extensively attempted for the synthesis of facile and economical metallic catalysts.

Rhodamine B is a widely used colorant in dyeing, textiles and food sectors, although it’s banned, but still practiced in food factories, and also a good water tracer fluorescent^11^. The carcinogenicity of RhB causes serious irritation of the skin, eyes and respiratory tract toward living beings^12^, so the development of a degradation system is always in demand to overcome water pollution issues by degrading organic pollutants. Many chemical, electrochemical, and photochemical ways, for example heterogeneous Fenton like photodegradation^13,14^, adsorption, microwave-assisted degradation, LED assisted catalysis and photodegradation with/without oxidants under different light sources, were practiced^15–21^. All afore-mentioned methodologies have some limitations such as usage of expensive materials, higher operational cost, longer degradation time and incomplete degradation.

The wide bandgap metallic oxides (CuO, TiO_2_) have presented splendid decolorization abilities in the UV light region (only 4% of the solar energy)^22,23^, but their extension to the visible light or near infra-red (IR) region is still a matter of great concern. The performance of a photocatalyst for photodegradation reaction is strongly depended on the number of active sites developed on the surface of the catalyst, light absorption ability (band gap), and the electron-holes (e^−^h^+^) generation/transfer efficiency of the catalyst^24^. Transition metals have great potential with their higher bandgap energy, (e^−^h^+^) generation and larger surface area for the degradation of organic matter.

Nanoparticles have some salient features such as simple and economical synthesis procedure, high surface area, good stability and easy recovery. These properties make them more favorable than other synthesis strategies of catalysts. Bimetallic nanoparticles have accomplished much attention because of their enhanced solid-state properties as well as they have better photocatalytic efficiency than their monometallic precursors^25^. Metals alloying over other semiconductor materials are consistently reported, in lieu of more active hetero-catalyst. Copper doped TiO_2_/polythiophene (PTh)^11^, Cu@Ag, Cu_2_O-Ag^26^, Cu-doped ZnO/ZnO heterostructures^27^, ZnO/CuO heterojunction^28^, CuCo_2_O_4_^29^, CuO nanowires and nanorods^23^, and Co_3_O_4_/Fe_2_O_3_^30^ were the different hybrid catalysts employed for the photodegradation reactions.

Copper and cobalt are two abundantly available semiconductor materials. Copper-based catalysts have excellent absorption properties in UV-region but cannot be practiced under visible light spectrum just because of their wide bandgap^23,31^. On the contrary, cobalt metallic centers have verified tremendous optical absorbance abilities in the visible light region, so, cobalt doping can be considered as a good tuner of the electro-optical properties of a bimetallic catalyst^32^. For instance, 3D continuous PPy@MnCo_2_O_4_/GNF nanoarchitectures and Co-Ni_3_S_2_@CNTs/GNF hybrid are Co doped composites, resulting in high specific capacitance alongwith excellent cycling stability for efficient HER electrocatalytic activity^33,34^.

UV-Vis/PS/heterogeneous photocatalysis is a relatively new, promising and the most effective technique for the removal of recalcitrant organic pollutants^35,36^, comparatively lower in operational cost and better photolysis abilities than the other oxidants such as PMS, PDS, per-iodate and Ce(NO_3_)_4_^37–40^. UV-Vis/PS oxidation process is successfully practiced in water oxidation reactions (WORs), degradation of p-chloroaniline and aniline, degradation of, phenol, propachlor atrazine^41–44^. The aforementioned catalysts had some limitations, such as unpractical pollutant degradation time (usually > = 100 minutes), narrow or precised pH, and higher catalyst cost upon noble metals coupling, to their commercial applications.

The prime objective of this work was the development of an optimum, simple, relatively cheaper and effective technique for the degradation of RhB dye. To achieve this goal, shuttle-like Co(OH)_2_/CuO nanoparticles were successfully synthesized by the simple co-precipitation method. Co(OH)_2_/CuO nanocomposite/PS system under visible light was successfully practiced for the removal of RhB dye within a short time interval for a broader pH range (pH 5–9) with excellent stability. Crucial parameters and recycling ability of Co(OH)_2_/CuO hybrid was also investigated. The reaction mechanism of Co(OH)_2_/CuO catalyst for the elimination of reluctant organic pollutant was also systematically explored.

## Results and Discussion

### Characterization of Co(OH)_2_/CuO

Crystallographic studies of as-synthesized catalysts were performed by XRD patterns. The sharpness of XRD clearly displays that the bimetallic catalyst has a highly crystalline structure. XRD patterns were then analyzed carefully by using JADE 6.5 software. Fig. S1. Supporting Information (SI), displayed five diffraction peaks located at 38.9, 39.2, 68.5, 72.2, and 83.6°, and these peaks were well indexed to diffraction of (111), (200), (220), (311) and (222) planes for CuO (reference: PDF#74-1021). For Co(OH)_2_ XRD peaks are located at 32.5, 58.1, 61.7, 68.2, and 81.3° and were well indexed to diffraction of (100), (110), (111), (200), (202) planes (reference: PDF#74-1057). With higher copper contents it was clearly observed that peaks of both metal oxides slightly shift to a higher 2θ angle. However, XRD patterns did not provide any clear indication about the presence of Co(OH)_2_, diffraction patterns only suggest that synthesized nanostructure is composite of CuO, but the XPS, ICP analysis and FESEM elemental mapping ensured the existence of a small amount of Co(OH)_2_ in the bimetallic catalyst.

FESEM images Fig. S2(a–d) SI, confirms that there are many nanoparticles in the resulting structure of as-synthesized bimetallic oxide having an approximate size of 500 nm. The morphology of Co(OH)_2_/CuO catalyst is shuttle-like and the approximate width and the length of nanoparticle was 30 nm and 80 nm respectively. In FESEM mapping, metallic distributions of Cu and Co were explored, and the results demonstrated that the amount of Cobalt in the bimetallic oxide is considerably low but both Cu and Co were evenly distributed (Fig. S3.SI). Morphological results of all other bimetallic oxides (different wt. ratios of Cu and Co) revealed by FESEM mapping analysis were also similar as of Co(OH)_2_/CuO catalyst (Figs. S4-3,S4-6.SI). HR-TEM analysis was also performed to confirm the morphology and existence of the contents of bimetallic oxide. Co(OH)_2_ was clearly observed, in the form of clusters, from lattice fringes along with CuO (Figs. S5-3. SI). HR-TEM analysis for other bimetallic oxides was also in a similar fashion as of Co(OH)_2_/CuO bimetallic oxide (Figs. S5-1, S5-2 and S5-4. SI).

**Figure 1:**
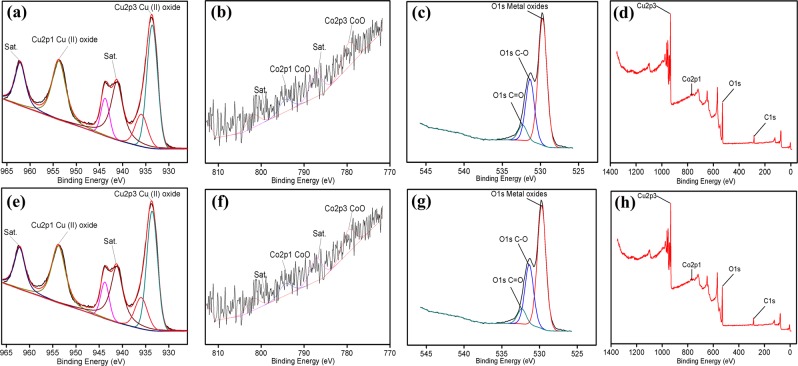
XPS of fresh (**a**–**d**) and recycled (**e**–**h**) Co(OH)_2_/CuO catalyst.

Figure 1(a–d) is the XPS analysis for the freshly synthesized of Co(OH)_2_/CuO catalyst. These results demonstrated the bonding configuration of both copper and cobalt metals and also provide information about the composition of the as-synthesized bimetallic catalyst. XPS analysis reveals that as-synthesized bimetallic oxide had followed the Cu 2p and Co 2p energy regions. The peaks at 933.8 eV and 953.7 eV were Cu 2p3/2 and Cu 2p1/2, respectively. The peaks at 779.6 eV and 793.9 eV were Co 2p3/2 and Co 2p1/2, respectively. These results proposed the existence of Cu (II) and Co (II). The O1s peak is clearly divided into three components (C–O, MO_x_ (M=Cu, Co) and C=O). Figure 1(e–h) is the XPS spectrum for the recycled and used catalyst, and, it can be seen clearly that there is no obvious shift observed in the peaks of Cu, Co, O for the fresh and recycled catalyst. XPS analysis for other bimetallic oxides was also founded similar to Co(OH)_2_/CuO bimetallic oxide (Figs. S4-1, S4-2, S4-4 and S4-5 SI). ICP-MS analysis of Co(OH)_2_/CuO catalyst illustrates that the Co contents are about 0.12% in the catalyst, demonstrating that there are traces of Co in the bimetallic catalyst. ICP-MS results revealed that the amount of copper and cobalt contents in Co(OH)_2_/CuO bimetallic catalyst were 774894 ppm and 937.80 ppm respectively (Table S1 SI). A surface area analysis of Co(OH)_2_/CuO catalyst has verified that the as-synthesized catalyst has excellent dye removal properties because it posed a high surface area (42.313 m^2^/g) (Table S2 SI).

Moreover, the results of XRD, FESEM mapping, ICP-MS and XPS, suggested that the compositions of Co(OH)_2_/CuO catalyst and all other bimetallic oxides studying in this work had exactly same compositions and configuration (ESI Fig. S1, Table S1) and Co(OH)_2_/CuO catalyst was selected for the further photodegradation experiments.

### Catalytic performance of Co(OH)_2_/CuO catalyst

The adsorption of organic molecules on the surface of photocatalysts had been verified to influence greatly the photocatalytic degradation of the organics^45^. To investigate adsorption and degradation mode, the catalytic performance of Vis/Ps/Co(OH)_2_/CuO catalyst based system was practiced by selecting (RhB) dye as a model pollutant. To ensure visible light only for degradation system, a cut filter of >400 nm was used. Initially, the catalyst was adsorbed in RhB solution for 20 minutes under dark conditions to investigate the effect of adsorption, after that, this system was subjected under Vis-light with the addition of PS for photo-decolorization. No obvious adsorption was observed, while for the case of photodegradation, Co(OH)_2_/CuO catalyst was found best amongst all other compositions so, for the rest of the study it will be described.

**Figure 2:**
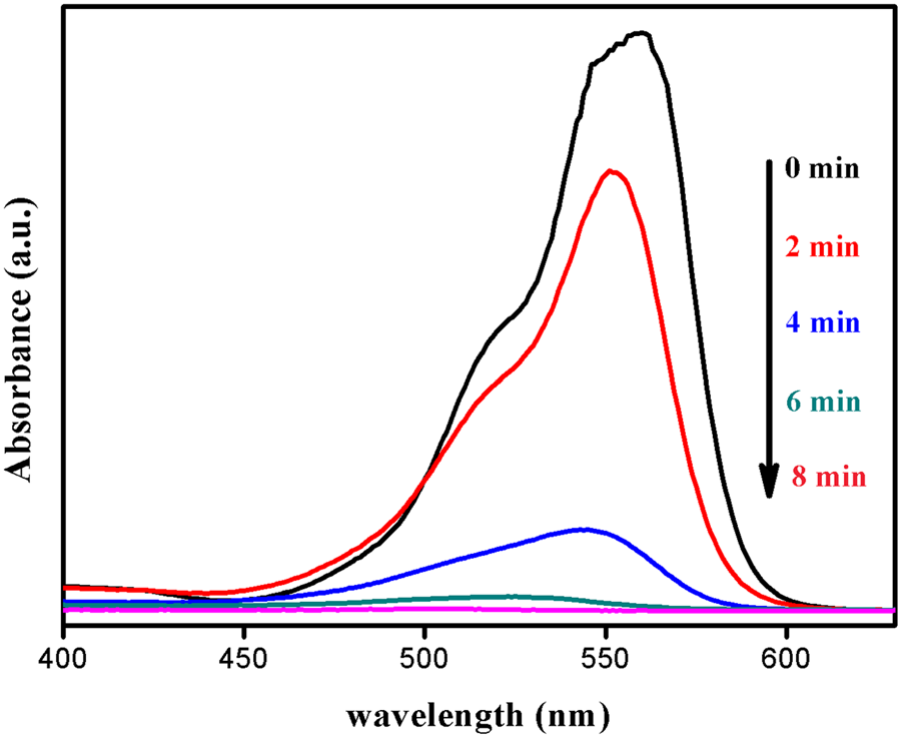
Spectroscopic study for degradation of Rhodamine B, other conditions are; reaction volume = 10 mL, initial pH = 7, PS dosage = 20 mg, initial dye concentration (D.C) = 100 mg/L and catalyst dosage (C.D) = 20 mg.

UV-Vis absorption spectrum in Fig. 2 revealed that decolorization for Vis/Ps/Co(OH)_2_/CuO based photocatalytic system was approached to 33.5% in 2 minutes under visible light illumination and after 8 minutes, dye concentration was approached to 0.0105 mg/L from 100 mg/L. In order to regenerate catalyst, the degraded solution was allowed to settle all the catalyst for two hours, after that, it was seperated and was named as R-Co(OH)_2_/CuO catalyst. Further, similar reaction systems for without Co(OH)_2_/CuO catalyst, without persulfate, and without light were also practiced.

### Performance optimization of Co(OH)_2_/CuO catalyst for photolysis reaction

The normalized concentration of degraded dye was studied by varying different parameters e.g. concentration of dye, the dosage of bimetallic catalyst, per-sulfate dosage, and the initial pH of the solution. Under optimized conditions binary metal oxide Co(OH)_2_/CuO catalyst has proved itself better than the single metal oxides (CuO, CoO and Co(OH)_2_) for photodegradation. This bimetallic oxide is also superior in its actions because of the crucial role played by Cu having extraordinary abilities to degrade many azo dyes. While on the other hand Co absorbs light and resulting in the generation of electron and hole pairs^1^, so, the combined effect of Cu and Co oxides posed better photo-decomposition activity.

**Figure 3:**
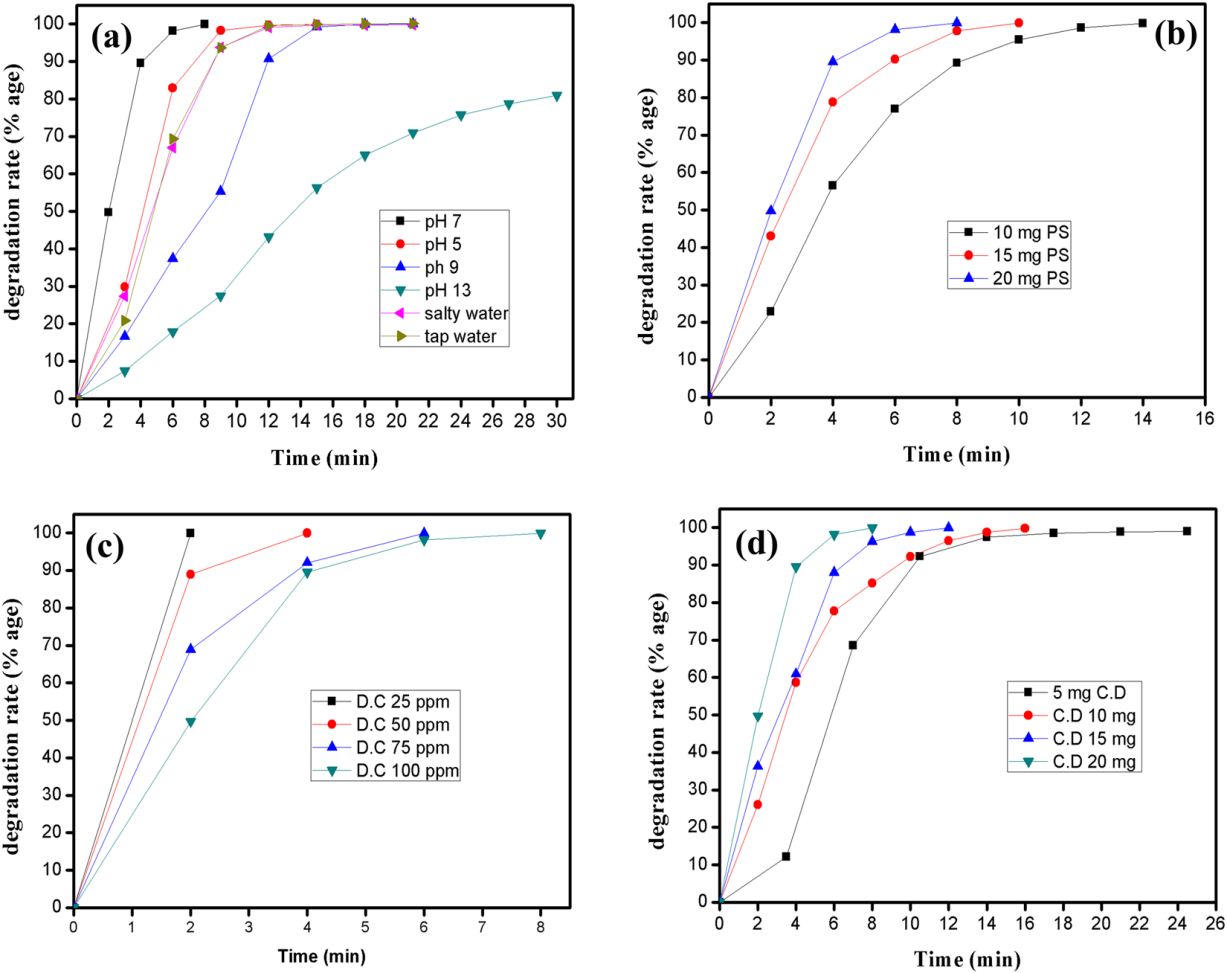
Effect of different parameters on RhB degradation under illumination; (**a**) effect of initial pH, (**b**) Effect of PS dosage, (**c**) effect of initial dye concentration (D.C) and (**d**) Effect of catalyst dosage (C.D).

Figure 3(a–d) illustrates the behavior of four key parameters, that are the initial pH of the system, PS dosage, initial dye concentration (D.C) and catalyst dosage (C.D), playing a vital role in photodegradation reaction. Four different initial concentrations (25, 50, 75 and 100 mg/L) were practiced during this study and 100 mg/L was selected for further studies. From 5 mg to 20 mg, four different catalyst dosages were studied and 20 mg was preferred for further studies as it had better degradation results in lesser time.

PS plays an important role in the photodegradation of organic pollutants by providing active sites and a platform for the exchange of electrons during reacting with catalyst, so, alike the absence of the catalyst, the absence of PS also did not permit photodegradation reaction. For optimum PS dosage, different persulfate quantities (10 mg, 15 mg and 20 mg) were studied and 20 mg PS dosage was observed as most suitable for further studies. Further increase in PS dosage may cause a reduction in organic matter degradation probably because of a faster and instantaneous generation of sulfate radicals (SO_4_^•−^) and they can react with each other to form per-sulfate as per following reactions^46^.

Usually, basic pH solutions or acidic pH solutions were reported for RhB degradation, as their catalysts had evidenced that their best performance was either in acidic or in basic media^47,48^. Therefore, different pH solutions were monitored to observe the effect of initial pH and fortunately Co(OH)_2_/CuO has performed well for a variety of pH (3–13) solutions. More specifically for neutral pH or the nearly neutral pH either slightly acidic (pH = 5) or slightly basic (pH = 9), bimetallic oxide had exhibited excellent photo-decolorization performance. But neutral pH was selected for further studies as it posed the best photo decolorization.

For photolysis, the bandgap energy of a catalyst allows the generation of the electron-hole pair at conduction and valence band respectively. Excitation of electron takes place from the valence band to conduction band when absorption of light takes place. In this work PLS-SXE300D, a high power xenon light source, with a cut filter of >400 nm, was used for the excitation of electrons, responsible for the generation of different radicals taking part in the photodegradation of RhB.

Figure 6S SI, explains a comparison of photo-degradation kinetics of different composites and also provide information about the role of per-sulfate and Co(OH)_2_/CuO catalyst when interacting with RhB individually. Under optimum conditions, CoO and Co(OH)_2_ had 13% and 9.13% catalytic activities for RhB degradation respectively, while both CuO and Co(OH)_2_/CuO catalyst displayed excellent activity with different degradation time. Almost 99.9% RhB was degraded with Co(OH)_2_/CuO catalyst by lowering RhB concentration from 100 mg/L to 0.0105 mg/L in presence of light, that is, obviously because of synergistic effect between CuO and Co(OH)_2_. Table S3 highlighted the performance of different photocatalysts and from results, it’s clear that bimetallic catalyst synthesized in this work had shown the best catalytic performance in a very short time as compared to other reported catalysts.

In order to highlight the unique optical properties of Co(OH)_2_/CuO, UV-Vis diffuse reflectance spectrum was carried out. The comparative results depicted in Fig. 7S(a) illustrated that the Co(OH)_2_/CuO catalyst possessed greater light absorbance than the all other catalysts, especially in visible light (400–600 nm) region. Similar results were recorded by performing photocurrent responses of different catalysts as a working electrode with the light switch on and off and shown in Fig. 7S(b), a work reported by our research group^49^. The photocurrent response of the Co(OH)_2_/CuO was considerably higher than the other catalysts under the same condition. The significant enhancement of the photocurrent response of Co(OH)_2_/CuO could be valuable for degradation studies. These results confirmed that Co(OH)_2_/CuO has good light absorption abilities with excellent photo-response.

Moreover, to evaluate the photocatalytic ability of Co(OH)_2_/CuO catalyst, the same reaction system was adopted and practiced, for two other most common organic pollutants i.e. Congo Red CR and Methyl Orange MO, as was adopted for RhB degradation. Results revealed that adsorption of Co(OH)_2_/CuO catalyst has no role in the degradation of CR and MO dyes and almost all the pollutants were photo-degraded efficiently and results suggested that the Co(OH)_2_/CuO/PS system under visible light is a highly efficient way to degrade many organic pollutants.

### Radical identification

**Figure 4:**
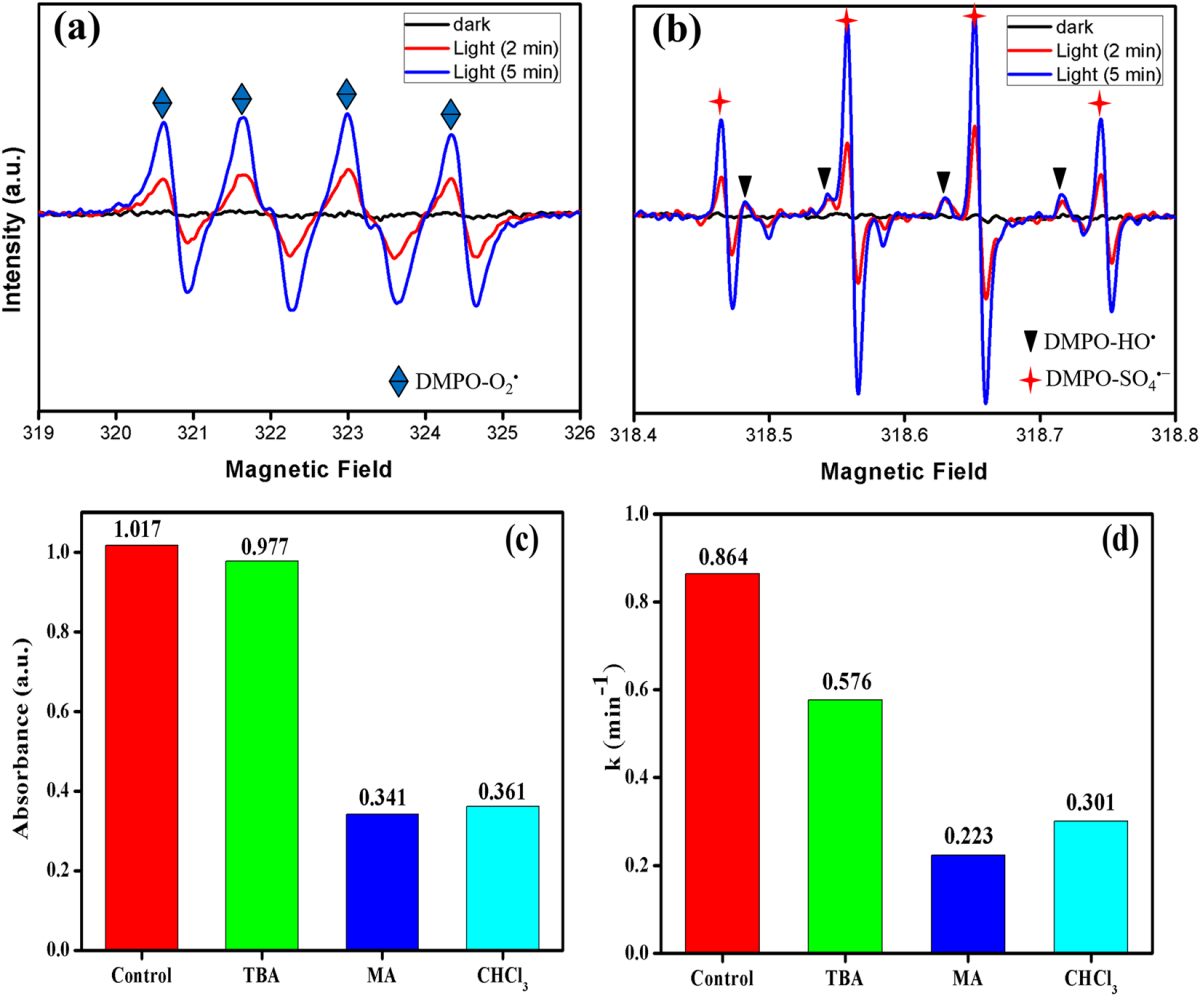
ESR spectra of radical adducts trapped by DMPO; (**a**) (O_2_^•^) & (**b**) (SO_4_^•−^, HO^•^) Radicals, Identification of active species. (**c**) Effect of different radical quenching reagents alongwith control experiment (**d**) Rate constant (Reaction conditions: Vol. 10 ml, Co(OH)_2_/CuO 20 mg; vigorous stirring at RT; TBA, CHCl_3,_ and MA dosages were 10 times equivalent of Na_2_S_2_O_8_).

The activation of sodium persulfate (PS) in the heterogeneous catalysis could produce SO_4_^•−^, HO^•^ and/or O_2_^•^ radicals. In order to identify the radicals generated during the photocatalytic reaction and their role in photocatalysis, electron spin resonance (ESR) technique was adopted, 5,5-Dimethyl-1-Pyrroline-N-Oxide (DMPO) was used as the radical capture agent. ESR results evidenced that sulfate (SO_4_^•−^), Superoxide (O_2_^•^) and hydroxyl (HO^•^) radicals were generated during Vis/Ps/Co(OH)_2_/CuO based photocatalysis system and SO_4_^•−^ and O_2_^•^ radicals were mainly responsible for the photocatalytic dye degradation. Figure 4(a,b) clearly demonstrated that the most dominant radical was SO_4_^•−^ radical while O_2_^•^ has an intermediate role in the photocatalysis process. The role of hydroxyl (HO^•^) in the photocatalytic reaction is almost negligible. The effect of light for activation of radicals generated from PS based heterogeneous catalysis was also highlighted by ESR spectra. Initially, under dark conditions, there were few radicals but when the catalytic system was exposed to light the radical’s generation was more and more with an increase in time.

The results claimed by ESR were also verified by introducing three different radical quenching reagents. Methanol (MA), Chloroform (CHCl_3_) and tert-butyl alcohol (TBA) were studied as quenching agents for SO_4_^•−^, O_2_^•^ and HO^•^ radicals respectively. Methanol (MA) with α-hydrogen was selected as probe agent for capturing both hydroxyl and sulfate radicals at significant rates (k SO_4_^•−^/_MA_ = 1.6–7.7 × 107 M^−1^ s^−1^, k HO^•^/_MA_ = 1.2–2.8 × 109 M^−1^ s^−1^), while, TBA without α-hydrogen was chosen as a probe agent for studying the reaction of hydroxyl radicals (k HO^•^/TBA = 3.8–7.6 × 108 M^−1^ s^−1^). On the other hand, CHCl_3_ was used as a scavenger for superoxide (O_2_^•^) radicals^50^. The role of these three radicals played during Vis/Ps/Co(OH)_2_/CuO based photocatalysis was also explored by these quenching reagents.

In the reaction system, all the radical scavengers were 10 times more concentrated than the PS concentration. Figure 4(c), clearly demonstrated that a significant difference was observed in the photolysis results of tert-butanol alcohol, chloroform, and methanol. RhB degradation was significantly reduced at the addition of methanol because it had breached photocatalytic reaction immediately on its addition to the reaction system by eliminating sulfate and hydroxyl radicals. At 10 equivalent addition of TBA, the photodegradation was slightly decreased (96%), the absence of α-hydrogen in TBA has a fewer effect on the generation of sulfate radicals, that’s why the photodegradation rate was not significantly influenced. At the addition of CHCl_3,_ the generation of superoxide radicals was inhibited, an ultimate reason to slow down the photodegradation process and as a result efficiency of the photocatalytic system was decreased to 64%. This claim was also evidenced by Fig. 4(d), as it was clearly demonstrated that, under controlled reaction conditions when there wasn’t any radical scavenger, the rate constant (k) was much higher than in the presence of any scavenger. Hence, as a result, it was verified that SO_4_^•−^ and O_2_^•^ radicals had a lead role in this photolysis system.

### Stability and recycling of catalyst

**Figure 5:**
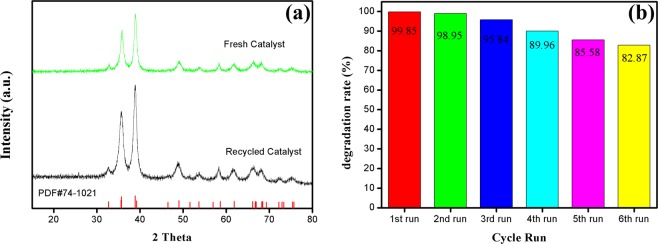
Recycle study of Co(OH)_2_/CuO catalyst (**a**) XRD analysis of fresh and used catalyst, (**b**) Percentage degradation for each cyclic run.

R- Co(OH)_2_/CuO catalyst was collected and characterized by XRD and XPS measurements. From Fig. 5(a) it is clear that the main diffraction peaks of CuO and Co(OH)_2_ for recycled catalyst have no obvious shift comparative to XRD patterns of fresh bimetallic oxide, indicating that Co(OH)_2_/CuO catalyst is highly efficient and stable during photolysis of RhB dye. X-ray energy spectrum (Fig. 1(e–h)) demonstrated that the peak pattern of the active metallic centers of the catalyst did not shift, indicating that the Co(OH)_2_/CuO catalyst was highly stable during the photocatalytic studies. For recycled Co(OH)_2_/CuO, even after the 6th run, the main and satellite peaks of Cu and Co were consistent with the main and satellite peaks of fresh Co(OH)_2_/CuO catalyst. The bimetallic catalyst synthesized in this work has high selectivity and reproduction abilities. Form Fig. 5(b), the stability and reusability of the Co(OH)_2_/CuO catalyst was verified for the photocatalytic reaction. The recycling of as-synthesized catalyst was observed in six successive runs to patterned its regeneration and sustainability.

Each cyclic test was performed for 8 minutes, that is basically overall reaction time for completion of reaction under optimum conditions for fresh catalyst, then for 2 hours let the resulting solution to settle all the catalyst, the catalyst was separated and was reused for next run with the addition of PS. After the sixth run, the catalytic activity of the catalyst was reduced from 99.9% to 82.87%. There are two possible reasons for this reduction in activity, first is basically related to the loss of some catalyst during the separation process and second is deposition of some carbon on the surface of the catalyst after each run, but still, 82.87% is an appreciable degradation efficiency within 8 minutes.

Leaching experiments were employed to estimate the stability of bimetallic catalyst and role of Cu^2+^ and Co^2+^ in the photolysis, the concentration of Cu^2+^ and Co^2+^ in leached-out Co(OH)_2_/CuO catalyst was analyzed by Atomic Absorption Spectrometry (AAS). Results illustrated that a very little amount of Cu^2+^ and Co^2+^ exuded from Co(OH)_2_/CuO catalyst i.e. 0.07% and 0.01% respectively. The supernatant with the leached-out Cu^2+^and Co^2+^ was first used to catalyze photolysis, for exploration of the relation of photolysis with the dissociation of Cu^2+^/Co^2+^. However, the results showed that no dye degradation was practiced by leached-out supernatant, which unveiled that Cu^2+^ & Co^2+^ has no participation for photocatalytic reaction.

### Mechanism and reaction pathway

**Figure 6:**
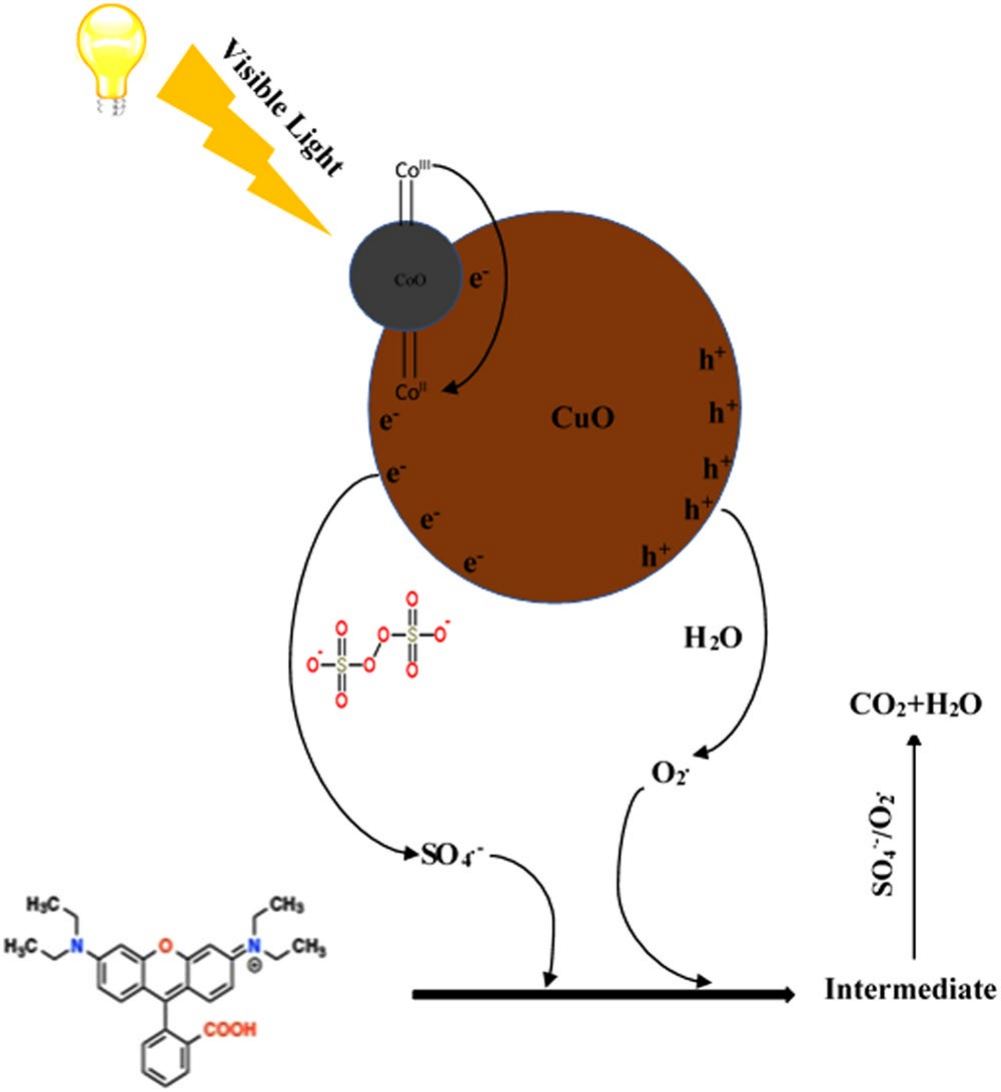
Purposed photocatalysis reaction mechanism for the degradation of RhB by Co(OH)_2_/CuO catalyst under visible light.

Heterogeneous semiconductor photocatalysis allows both reduction and oxidation state to take place at the same time on its surface. The PS based mechanism for activating heterogeneous catalysts have been broadly studied in water treatment^51^. The mechanism of heterogeneous photocatalysis can be explained by the Langmuir–Hinshelwood kinetic technique i.e. basically the simultaneous generation of electrons (e−) and holes (h+) under light irradiation. These holes are then trapped by the catalyst surface absorbed organic pollutants for the production of a radically reactive state which was recombined with a photogenerated electron to decompose the pollutant. Subsequently, the regeneration of catalyst takes place that can be further used^52^. A schematic illustration of a possible mechanism for photodegradation of rhodamine B by activating Co(OH)_2_/CuO catalyst bimetallic catalyst with PS under visible light was proposed as shown in Fig. 6. A similar illustration was already reported with SO_4_^•−^ and HO^•^ as dominant species in their Fenton like reaction system^47^.

At first, the Co(OH)_2_ was excited under visible light irradiation and generated photoexcited electrons (e^−^) and holes (h^+^). These photoexcited electrons contributed to decomposition reaction by reacting with PS to generate SO_4_^•−^ radicals having a major contribution to degradation reaction. While holes reacted with H_2_O to produce superoxide (O_2_^•^) radicals. In photocatalytic reaction superoxide (O_2_^•^) radicals although posed less role than sulfate SO_4_^•−^ radicals but still playing a vital role in the photodegradation of RhB. Such cyclic production of these radicals progressively increases the photocatalytic degradation activity of bimetallic catalysts towards organic pollutants.

The reaction pathway was also systematically explored by using HPLC-MS. Mainly six intermediates (A-F) were found to be involved by HPLC-MS chromatograms, at different retention times during RhB decolorization with vis/Ps/Co(OH)_2_/CuO based photocatalyst. From the results as tabulated in Table S4, it’s clear that first peak i.e. A at retention time (t_R_) 9.8 min purely belongs to RhB, while peak B (t_R_ 11.6 min), C (t_R_ 13 min), and D (t_R_ 13.3 min) were species identified as N-de-ethylated intermediates in positive (+ive) ion mode, while derivative of RhB at peak E (t_R_ 19.2 min) and H (t_R_ 22 min) were identified as benzoic acid, phthalic acid were in negative (−ive) ion mode. The structural formulae of N-de-ethylated intermediates with their accurate mass and M^+^ peaks were also discussed in Table S4. The rapid decrease in the intensity of peak A (RhB) can be observed after a reaction time of 2 min, on contrary, there was an increase in the peak of intermediates (B–E) initially but decreased shortly due to their conversion into F intermediate.

**Figure 7:**
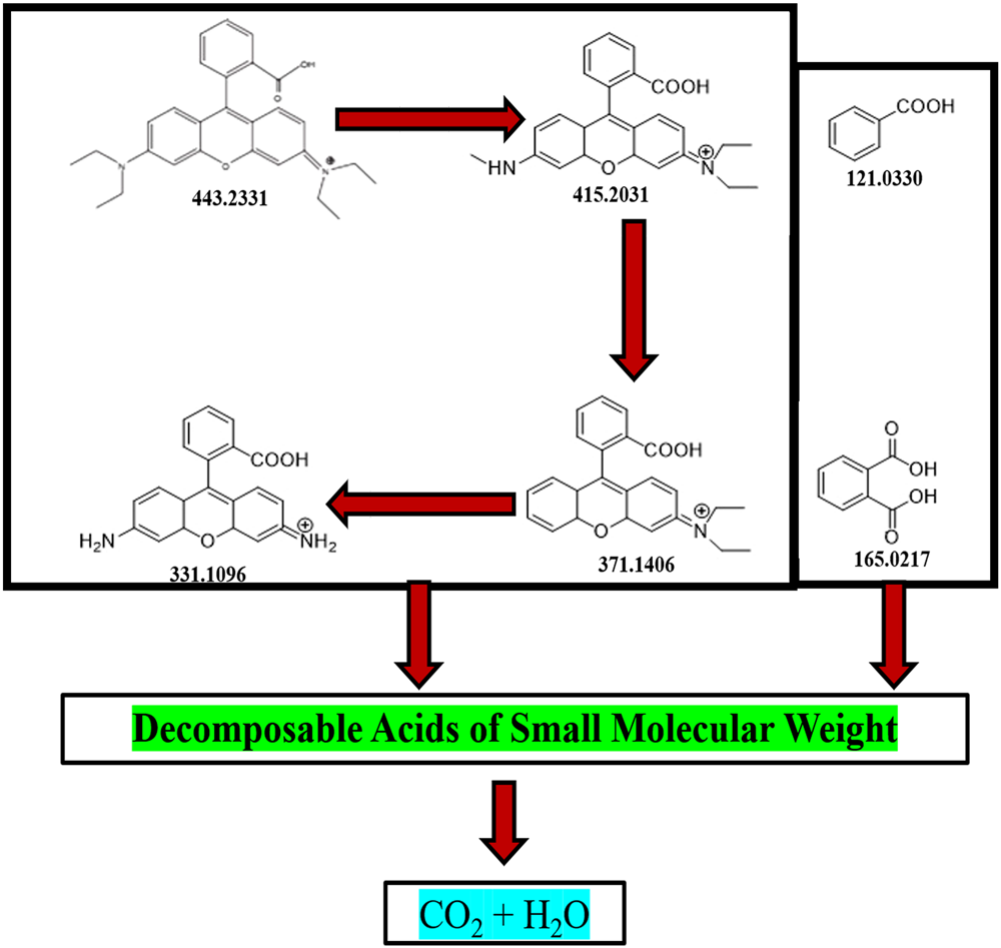
Proposed reaction pathway for RhB degradation under Vis/Ps/Co(OH)_2_/CuO based system.

Peak F also got some gain initially but this cleavage was broken under continuous light irradiation as reaction time approached to 8 minutes. A-F intermediates underwent ring-opening and mineralization, as a result, the production of biodegradable small molecular acids took place. N-de-ethyl group on the dye molecule was immediately approached by the SO_4_^•−^ and O_2_^•^ radicals, as a result, ring-opening and mineralization occur and subsequently, it led to the formation of (A-F) intermediates. Ring-opening step further resulting in the formation of some small molecular weighted biodegradable acids under continuous reaction. The ring-opening and partial mineralization pattern can be verified by many reported works such as recently reported work of Wenxing Chen and co-workers^53^. On the basis of HPLC-MS results, the possible degradation pathway of organic dye was also proposed as shown in Fig. 7.

**Figure 8:**
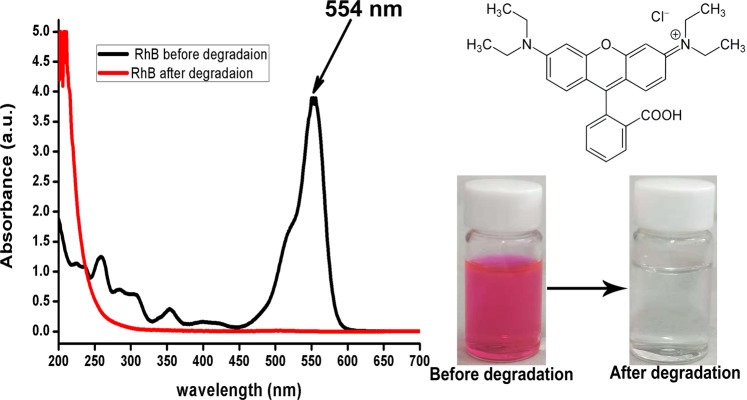
Structure/Properties of Rhodamine B before and after degradation reaction.

For the determination of complete degradation of RhB, the degraded product was analyzed by using gas chromatograph (GC), at different retention times. Figure 8S(a) illustrates that decomposed products comprise of CO_2_ gas with a gradual increase observed until the reaction was over. Resulted CO_2_ product was also verified by comparing it with pure CO_2_ gas and both analyzed peaks were consistent to each other as shown in Fig. 8S(b). On the basis of HPLC and GC results, it can be verified that generation of CO_2_ gas taken place but complete degradation cannot be predicted, definitely, there will be some partially degraded by products alongwith CO_2_ and H_2_O that’s why a partial degradation has been concluded.

## Conclusion

Co(OH)_2_/CuO nanocomposite was prepared by a simple co-precipitated process and was examined by different analytical techniques. Co(OH)_2_/CuO catalyst was optically active, having the synergic effect of cobalt hydroxide modification on copper oxide, and posed excellent potential application towards organic pollutants degradation in the visible light region. This work mainly emphasizes the performance of newly synthesized Co(OH)_2_/CuO catalyst and effect of initial pH of the solution on the photocatalysis reaction and it’s an achievement to degrade RhB not just in under neutral pH but also for basic or acidic pH (5–9) in such a shorter time with Co(OH)_2_/CuO/PS/Vis-Light based system. Mechanistic studies had explored that sulfate radicals and superoxide radicals were the key radicals responsible for the photolysis of RhB. HPLC and GC results suggested that partial degradation of pollutant into CO_2_ and water tookplace. Most importantly, the regeneration of bimetallic catalyst is quite facile and have excellent (82.87%) recyclability even after six consecutive runs. The XRD and XPS measurements of R-Co(OH)_2_/CuO catalyst strongly recommend that the catalyst posed good stability. Also, Co(OH)_2_/CuO catalyst has verified its tendency for the degradation of many organic pollutants.

Thus, conclusively this bimetallic nanocomposite photocatalyst can be highly recommended for accomplishing photocatalysis in favor of health observations and polluted water resources, by keeping in view its simple synthesis procedure and less degradation time for the decomposition of toxic organic matters.

## Experimental Methods

### Chemicals

Na_2_S_2_O_8_ was purchased from ACROS. Rhodamine B (RhB) was purchased from Aladdin. NaOH, Na_2_CO_3_, NaCl, CHCl_3_, acetic acid, methanol (MA), tert-butyl alcohol (TBA), Cobalt (II) acetate tetrahydrate Co(CH_3_COO)_2_·4H_2_O, and Copper (II) acetate monohydrate Cu(CH_3_COO)_2_·H_2_O were supplied by Adams and Sigma. All the chemicals were used as received, without any further processing.

### Synthesis of Co(OH)_2_/CuO bimetallic nanoparticles

A simple co-precipitation method was adopted for the synthesis of Co(OH)_2_/CuO catalyst in this work. Typically, 0.360 g of Co(CH_3_COO)_2_·4H_2_O and 1.20 g of Cu(CH_3_COO)_2_.H_2_O was weighed in a three-neck round bottom flask and were dissolved in 300 mL deionized water (DI H_2_O). when the solution was completely salt-free then 1 ml glacial acetic acid (CH_3_COOH) was added, followed by the addition of 0.8 g sodium hydroxide (NaOH). The solution was refluxed at 100 °C for 1 hour to allow the reaction to be completed. Brownish black precipitates were formed after the completion of the reaction. Obtained precipitates were first cooled down to room temperature, then centrifuged, washed once with DI water and were dried in a vacuum oven at 60 °C over-night The catalyst was named as Co(OH)_2_/CuO. CuO, Co(OH)_2_ and two different compositions of Co(OH)_2_/CuO catalysts named as Co(OH)_2_/CuO-1, and Co(OH)_2_/CuO-2 were also synthesized by varying cobalt concentration via similar co-precipitation methods.

### Synthesis of CoO nanoparticles

CoO nanoparticles were prepared by modifying the synthesis method reported by Wu, J. *et al*.^54^. Typically, 0.1 M Na_2_CO_3_ and 0.1 M Co(CH_3_COO)_2_·4 H_2_O solutions were prepared, each in 300 mL DI water. Both the solutions were mixed stirred for 30 mins until pinkish-purple precipitates appeared. Let the product naturally cool down to room temperature, centrifuged, washed 3 times with DI water and then overnight dried at 60 °C. Ground the dried product and calcinate it in an inert (N_2_) environment, at 400 °C for 2 hours with a heating rate of 10 °C.min^−1^. Black fine nanoparticles of CoO were formed.

### Characterization

To observe the morphology of samples FESEM and TEM techniques were employed. Field emission Scanning Electron microscopy along with mapping was analyzed by using a Hitachi SU8010 microscope performed at 5 kV. While Transmission electron microscopy was analyzed by using Hitachi-600 with an accelerating voltage of 200 kV. The crystal structure analysis and elemental compositions were performed by PXRD and XPS techniques respectively. Powder X-ray diffraction data was attained by using Rigaku D/max-ga X-ray diffractometer at a scanning rate of 6° min^−1^ while 2θ ranging from 5° to 80° with Cu K radiation (1.54178 Å). For peaks analysis, Jade 6.5 software was utilized. X-ray photoelectron spectroscopy was analyzed by using a Thermo Fisher Scientific XPS ESCALAB 250Xi instrument with an Al K (1486.8 eV) X-ray source. For UV-Visible absorption spectrum studies, the bimetallic catalyst was analyzed at Unico 4802S UV/VIS-NIR double beam spectrophotometer by using BaSO_4_ as reference material. Elemental analyses of Cu and Co were performed on an ICP-4300DV ICP atomic emission spectrometer. The concentration of copper ion was measured by Atomic Absorption Spectrometry (AAS) on Agilent 240 FS AA. Accelerated surface area calculations and porosimetry of Co(OH)_2_/CuO catalyst were carried out at autosorb iQ. Elemental analyses of Cu and Co were also performed on the ICP-4300DV ICP atomic emission spectrometer. The electron spin resonance (ESR) measurements were carried at JES FA300 Spectrometer using 5, 5-Dimethyl-1-Pyrroline-N-Oxide (DMPO) as the radical capture agent. HPLC-MS was carried out for the determination of intermediates, resulting in degradation of RhB, and also provide information for a proposed reaction pathway. Shimadzu GC-2014 was used for the detection of amount of CO_2_ generated at different retention times.

### Evolution of catalytic activity

The catalytic activity of Co(OH)_2_/CuO was observed at the Shimadzu UV-2550 spectrophotometer. The model organic matter studied in this study was RhB. A stock solution of 100 ppm concentration of RhB was prepared and the initial pH of the solution was neutral during the whole study. The optimum amount of as-prepared catalyst was added in 50 ml of 100 ppm RhB solution in a flask along with the predetermined amount of sodium persulfate. A xenon light source (PLS-SXE300D) was utilized in photo-decolorization studies and after every two minutes retention time of photo illumination, a sample of 3 ml was extracted, filtered by 0.22 µm membrane and then analyzed by UV-Vis spectroscopy (λ_max_ = 554 nm) until all the organic matter was decolorized.

After this, the final concentration of each sample was determined from the absorption spectrum and degradation rate was calculated by the expression 1,

1 $$degradation\,rate=\frac{{C}_{i}-{C}_{f}}{{C}_{i}}\,\ast \,100$$

where, *C*_*i*_ is referred to initial concentration while *C*_*f*_ is referred to final concentration of dye after each degradation interval. Figure 8 described the structure of RhB, highlighting the color of RhB before and after degradation and RhB absorption peaks before and after degradation. After complete degradation used catalyst was collected and denoted as R-Co(OH)_2_/CuO. RhB photocatalytic degradation process approximately followed pseudo-first-order kinetics and can be expressed as Eq. (2):

2 $$-\,\mathrm{ln}(\frac{{[RhB]}_{f}}{{[RhB]}_{i}})={\rm{Kt}}$$

where RhB_f_ and RhB_i_ are final and initial concentrations respectively before and after degradation and K (min^−1^) is reaction rate constant.

### Ethical approval and informed consent

All ethics were obeyed, all the experiments are in accordance with the guidelines.

## Supplementary information

Supplementary information

## Data Availability

No data available for this work, however, data can be provided on request.
