# Supplementary material for: Synergistic Catalysis of Co(OH)2/CuO for the Degradation of Organic Pollutant Under Visible Light Irradiation
Source: Sci Rep. 2020 Feb 6;10:1939. doi: 10.1038/s41598-020-59053-9 (PMC7005304; doi:10.1038/s41598-020-59053-9)
Supplement: Supplementary file 1 — Supplementary information [file 41598_2020_59053_MOESM1_ESM.pdf]

Synergistic Catalysis of $Co(OH)_2/CuO$ for the Degradation of Organic Pollutant Under Visible Light Irradiation

Akram Naeem,[1] Jia Guo,[1] Wenlan Ma,[1] Yuan Guo,[1] Hassan Afaq,[1] and Jide Wang[*1]*

[1] Key Laboratory of Oil and Gas Fine Chemicals, Ministry of Education & Xinjiang Uygur Autonomous Region, College of Chemistry and Chemical Engineering, Xinjiang University, Urumqi, 830046, China.

[*] Corresponding author. *E-mail:* awangjd@sina.cn

Electronic Supporting Information:

Fig S1:

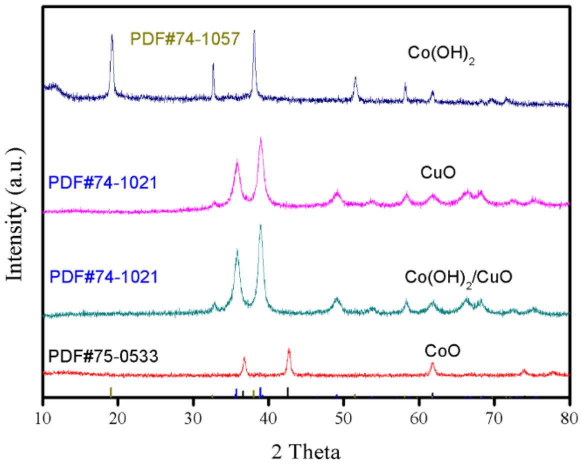

Fig. S1. XRD patterns of Fresh $Co(OH)_2$, CuO, $Co(OH)_2/CuO$ catalyst and CoO

Fig S2:

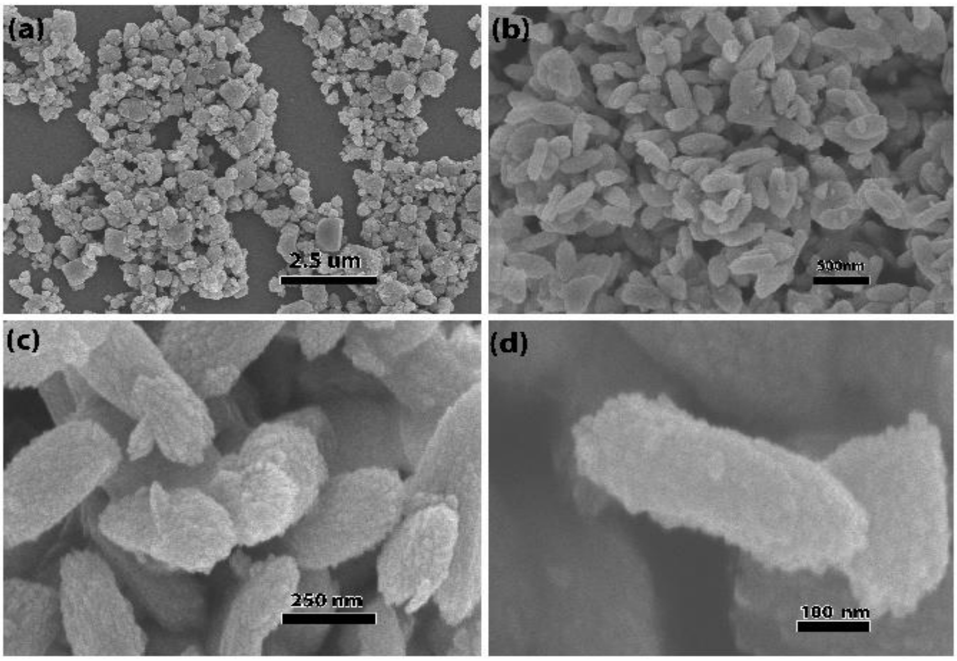

Fig. S2: FESEM Images for $Co(OH)_2/CuO$ catalyst (a-d)

Fig S3:

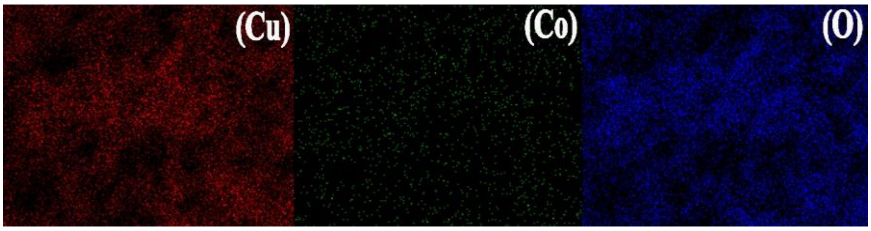

Fig. S3: FESEM mapping images of $Co(OH)_2/CuO$ catalyst

Table S1: ICP-MS Analysis

Sample	Cu	Co	$m_{Cu} : m_{Co}$	$n_{Cu} : n_{Co}$
	ppm	ppm		
$Co(OH)_2/CuO$ catalyst -1	783247	620.789	1261.696	1170.106
$Co(OH)_2/CuO$ catalyst	774894	937.797	826.29183	766.3088
$Co(OH)_2/CuO$ catalyst -2	767437	1993.89	384.89435	356.9537

Table S2: Surface Area analysis

Sample	Surface Area	Pore Volume	Pore Radius
	m^2g^{-1}	cc/g	nm
$Co(OH)_2/CuO$ catalyst	42.313	0.088	1.7025

Fig S4:

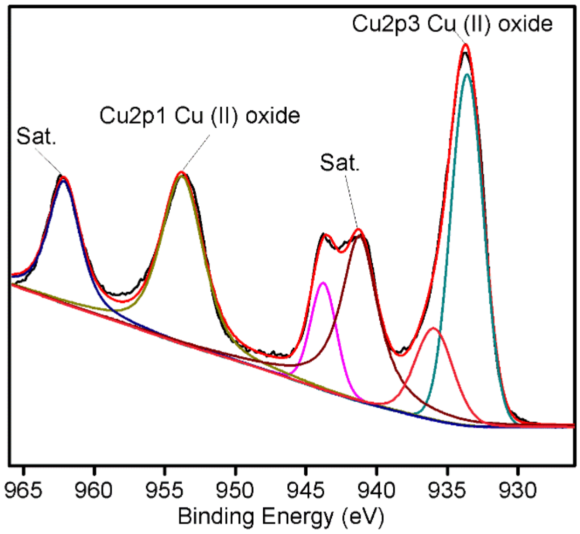

Fig. S4-1 XPS of $Co(OH)_2/CuO$ catalyst -1 in the energy regions of Cu 2p.

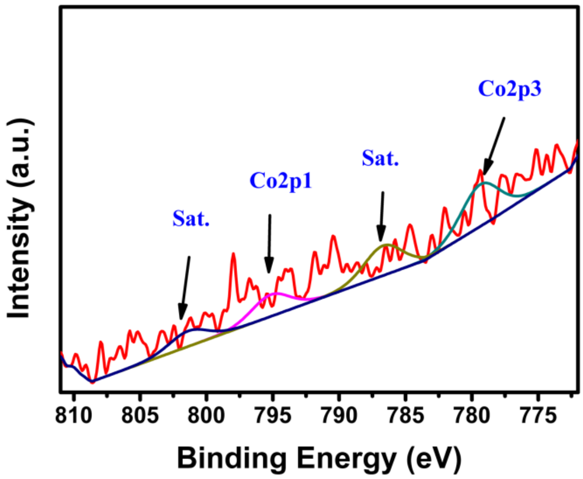

Fig. S4-2 XPS of $Co(OH)_2/CuO$ catalyst -1 in the energy regions of Co 2p

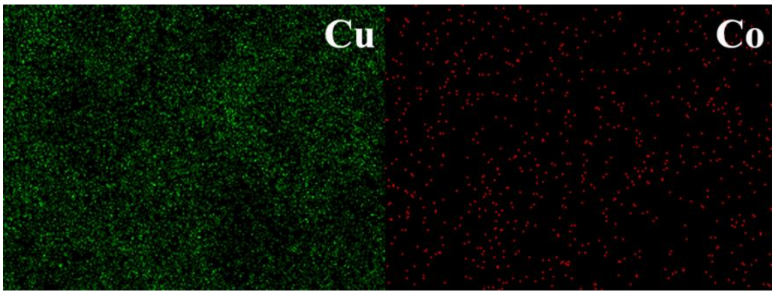

Fig. S4-3 FESEM mapping images of $Co(OH)_2/CuO$ catalyst-1

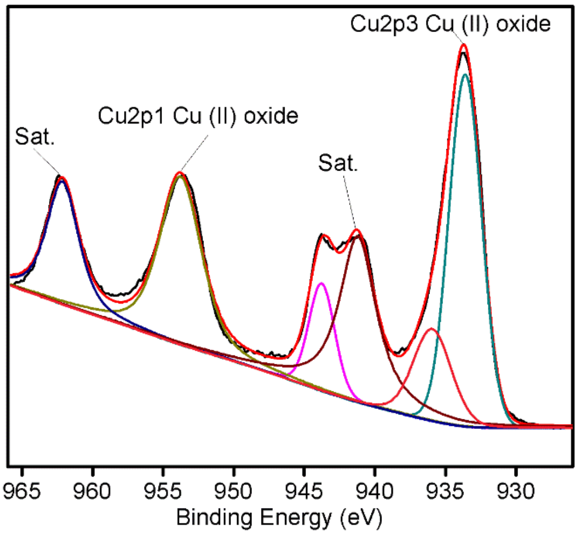

Fig. S4-4 XPS of $Co(OH)_2/CuO$ catalyst -2 in the energy regions of Cu 2p

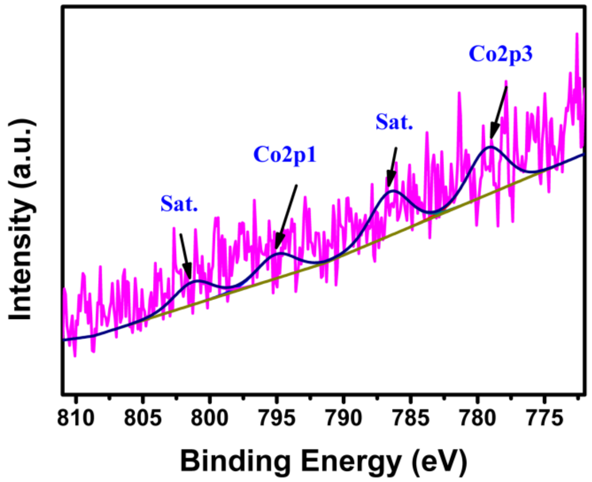

Fig. S4-5 XPS of $Co(OH)_2/CuO$ catalyst -2 in the energy regions of Co 2p

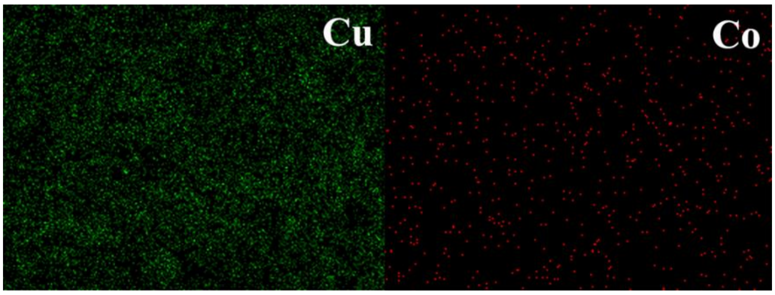

Fig. S4-6 FESEM mapping images of $Co(OH)_2/CuO$ catalyst-2

Fig S5: TEM analysis for $Co(OH)_2$ modified CuO catalyst

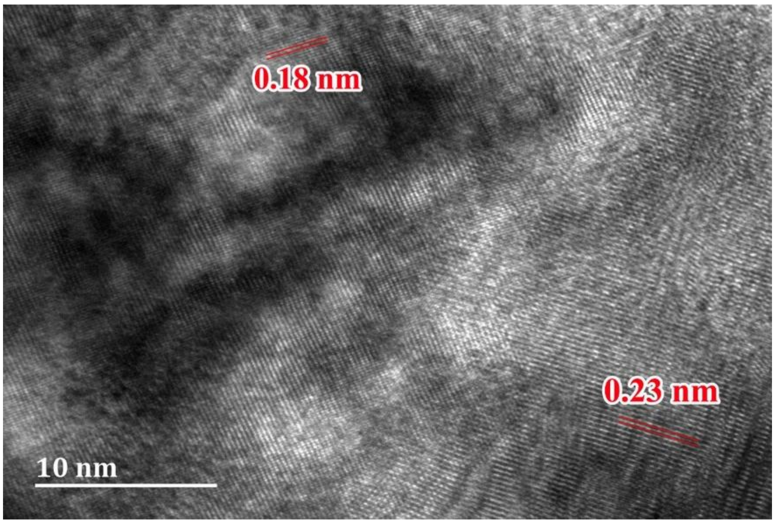

Fig. S5-1 TEM images of $Co(OH)_2/CuO$ catalyst -1

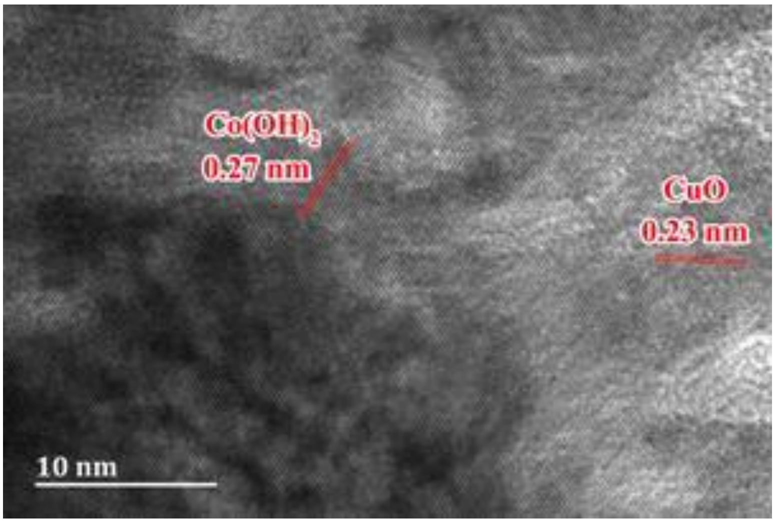

Fig. S5-2 TEM images of $Co(OH)_2/CuO$ catalyst -2

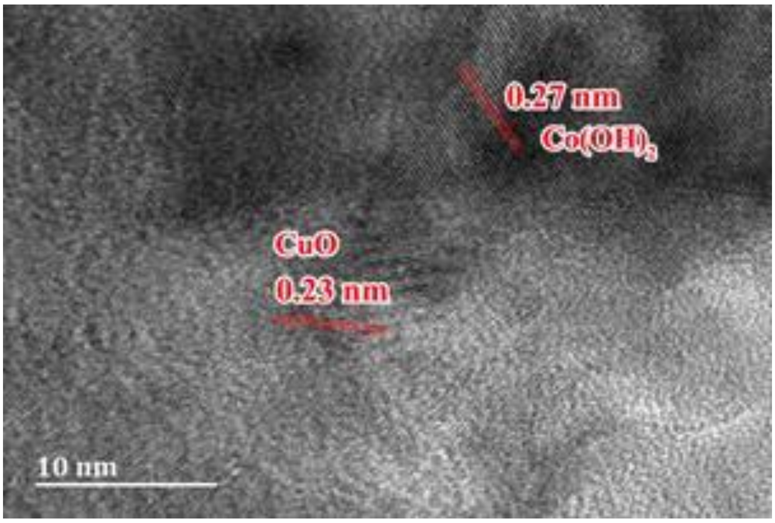

Fig. S5-3 TEM images of $Co(OH)_2/CuO$ catalyst

Fig S6:

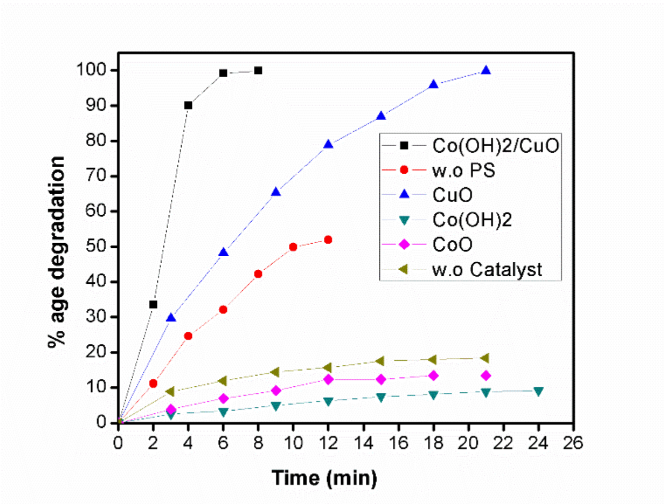

Fig S6: Photodegradation performance of CoO, CuO, $Co(OH)_2/CuO$ catalyst (with and without PS) and $Co(OH)_2$

Table S3: Comparison of the degradation rate of different bimetallic catalysts.

Catalysts	Time (mins)	RhB Degradation (%)	Reference
$Co(OH)_2/CuO$	8	99.9	This work
CuO	21	99.9	This work
CoO	21	13	This work
$Co(OH)_2$	24	9.13	This work
CuO Nanowires/Rods	280	85	[1]
$SiO_2@TiO_2$	480	99	[2]
OMC-CuO	100	97.2	[3]
TiO_2/ZnO Supported Au	160	98	[4]
ZnO/CuO	400	99	[5]

Fig S7:

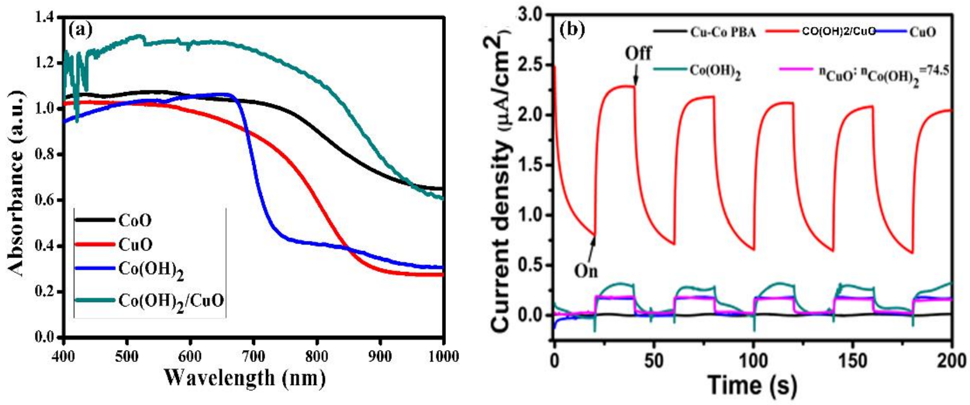

Figure 7S: (a) UV-Vis diffuse reflectance spectra of different catalysts (b) Transient photocurrent response of different samples; (Reproduced; Ref # [6])

Table S4: Detected species of RhB in the presence of CuO/Co(OH)$_2$ under visible light, examined by HPLC-MS.

Intermediate products	Retention time (min)	Mode	Formula	Measured accurate mass (m/z)	M^+ (m/z)
A	9.8	Positive	$C_{28}H_{31}N_2O_3$	443.2331	413.192<br>399.173<br>385.167<br>371.165<br>355.051
B	11.6	Positive	$C_{26}H_{27}N_2O_3$	415.2014	385.179<br>371.142<br>357.545<br>343.106
C	13	Positive	$C_{25}H_{27}N_2O$	371.1406	341.168<br>327.152
D	13.3	Positive	$C_{20}H_{15}N_2O_3$	331.1096	314.0843<br>287.1189<br>285.1029
E	19.2	Negative	$C_7H_6O_2$	121.0289	107.8182<br>92.095
F	22	Negative	$C_8H_6O_4$	165.0184	122.031<br>129.9847

Fig S8:

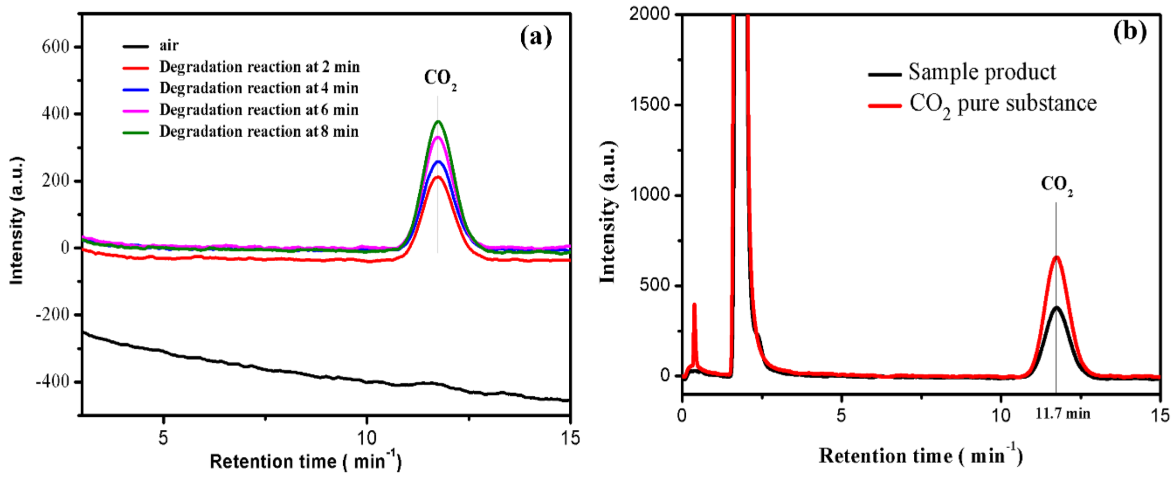

Fig S8: GC analysis for detection of CO_2 evolved during photocatalytic reaction of RhB; (a) analysis of decomposed product at different retention times (b) Comparison of pure CO_2 and produced CO_2 during photodegradation reaction

Reference:

1. Li, H., Liao, J. & Zeng, T. A facile synthesis of CuO nanowires and nanorods, and their catalytic activity in the oxidative degradation of Rhodamine B with hydrogen peroxide. *Catalysis Communications*. **46**, 169-173 (2014).
2. Wilhelm, P. & Stephan, D. Photodegradation of rhodamine B in aqueous solution via SiO2@ TiO2 nano-spheres. *Journal of Photochemistry and Photobiology A: Chemistry*. **185**, 19-25 (2007).
3. Tuerdi, A., Abdukayum, A. & Chen, P. Synthesis of composite photocatalyst based on the ordered mesoporous carbon-CuO with high photocatalytic activity. *Materials Letters*. **209**, 235-239 (2017).

4. Alshammari, A., Bagabas, A. & Assulami, M. Photodegradation of rhodamine B over semiconductor supported gold nanoparticles: The effect of semiconductor support identity. *Arabian Journal of Chemistry* (2014).
5. Pal, S., Maiti, S., Maiti, U. N. & Chattopadhyay, K. K. Low temperature solution processed ZnO/CuO heterojunction photocatalyst for visible light induced photo-degradation of organic pollutants. *CrystEngComm.* **17**, 1464-1476 (2015).
6. Guo, J., et al., Efficient difunctional photocatalyst prepared in situ from Prussian blue analogues for catalytic water oxidation and visible-light absorption. Catalysis Science & Technology. **8(24)**, 6375-6383 (2018).
